# Halitosis and *Helicobacter pylori* Infection: A Systematic Review and Meta‐Analysis

**DOI:** 10.1002/jgh3.70483

**Published:** 2026-10-11

**Authors:** Yu Xia, Jun‐Da Zhu, Tong Su, Bing‐Jie Jin, Shu‐Lei Zhao, Hong‐Wei Xu, Chang‐Qin Xu

**Affiliations:** ^1^ Department of Gastroenterology Shandong Provincial Hospital Affiliated to Shandong First Medical University Jinan Shandong China

**Keywords:** *H. pylori* eradication, *H. pylori* infection, halitosis, *Helicobacter pylori*, meta‐analysis

## Abstract

**Background:**

Halitosis, a common clinical condition, is frequently associated with *Helicobacter pylori* infection. Numerous studies have highlighted a significant reduction in halitosis following successful *H. pylori* eradication.

**Aim:**

This meta‐analysis aimed to investigate the association between *H. pylori* infection and halitosis, as well as the effect of *H. pylori* eradication on halitosis resolution.

**Methods:**

We searched major databases, including PubMed, EMBASE, the Cochrane Library, and the Science Citation Index, up to March 2025. Literature screening and data extraction were conducted according to strict inclusion and exclusion criteria. Study quality was assessed using appropriate tools according to study design. Heterogeneity and risk of bias were analyzed using Stata 12.0. Subgroup analyses were performed to examine the relationship between *H. pylori* infection and halitosis, including infection prevalence, symptom incidence, and treatment outcomes.

**Results:**

Of the 154 articles screened, 24 met the inclusion criteria, involving 5872 patients. The analysis revealed a significantly higher *H. pylori* infection rate in the halitosis group compared with the non‐halitosis group (OR = 3.42; 95% CI: 1.13–10.33). The prevalence of halitosis was higher in *H. pylori* ‐positive individuals than in *H. pylori* ‐negative individuals (OR = 1.91; 95% CI: 1.28–2.86). The prevalence of halitosis was lower in the successful eradication group compared with the unsuccessful eradication group (OR = 0.17; 95% CI: 0.04–0.73). Moreover, pre‐eradication halitosis rates were higher than post‐eradication rates (OR = 4.39; 95% CI: 1.99–9.68).

**Conclusion:**

The findings suggest a significant observational association between *H. pylori* infection and halitosis, and eradication therapy may be associated with alleviation of halitosis.

## 1 Introduction

Halitosis, characterized by abnormal odors emanating from the oral or nasal cavity, is a prevalent condition affecting individuals across all age groups [1]. It is estimated that approximately 50% of adults worldwide experience halitosis, with a significant occurrence also observed in children [2, 3]. Beyond its negative impact on social interactions and mental well‐being, halitosis may signal underlying pathological conditions. Halitosis is categorized into two main types: intraoral and extraoral [1]. Intraoral halitosis accounts for 80%–90% of cases, primarily caused by volatile sulfur compounds (VSCs) such as hydrogen sulfide (H_2_S), methyl mercaptan (CH_3_SH), and dimethyl sulfide (CH_3_SCH_3_), which are metabolic byproducts of oral bacteria [4]. Key contributing factors include tongue coating, periodontal disease, and gingivitis [5]. Extraoral halitosis, on the other hand, is often linked to systemic disorders like gastrointestinal diseases, respiratory infections, metabolic conditions, or hepatic/renal dysfunction [6, 7]. Accurate diagnosis of the underlying etiology is crucial for developing targeted therapeutic strategies.

*Helicobacter pylori* , a chronic bacterial infection with a global prevalence of approximately 50%, is strongly associated with conditions such as gastritis, peptic ulcers, and gastric cancer [8, 9]. Recent evidence suggests a potential link between *H. pylori* infection and halitosis. A 2016 meta‐analysis indicated that *H. pylori* infection might be correlated with halitosis, with eradication therapy providing symptomatic relief in refractory cases [10]. However, recent studies, including those by Yang et al., have reported no significant difference in *H. pylori* positivity rates between halitosis and non‐halitosis groups [11]. This study systematically reviewed high‐quality English literature and conducted a meta‐analysis to assess whether *H. pylori* infection is associated with halitosis and whether eradication therapy effectively alleviates symptoms, thereby offering evidence‐based guidance for clinical practice.

## 2 Methods

This systematic review and meta‐analysis was registered in PROSPERO (registration number: CRD420261327983; registered on February 27, 2026).

### 2.1 Data Sources and Search Strategy

A systematic search of major databases (PubMed, EMBASE, MEDLINE, and Web of Science) was conducted up to March 2025 to identify articles investigating the association between halitosis and *H. pylori* . The detailed search strategy for PubMed was as follows: (“Halitosis”[MeSH] OR “Halitosis”[Title/Abstract] OR “Halitoses”[Title/Abstract] OR “bad breath”[Title/Abstract] OR “oral malodor”[Title/Abstract]) AND (“ *Helicobacter pylori* ”[MeSH] OR “ *Helicobacter pylori* ”[Title/Abstract] OR “ *H. pylori* ”[Title/Abstract] OR “ *Campylobacter pylori* ”[Title/Abstract] OR “ *Campylobacter pyloridis* ”[Title/Abstract]). Similar strategies were applied to EMBASE, MEDLINE, and Web of Science.

Initial screening of titles and abstracts excluded records that did not meet the inclusion criteria or were deemed irrelevant to the research focus. Additionally, the reference lists of selected articles were reviewed to identify further relevant studies.

### 2.2 Inclusion and Exclusion Criteria

This study implemented rigorous screening criteria: *H. pylori* positivity required confirmation by at least two validated methods (urea breath test, histology, or PCR). Halitosis diagnosis required objective measurement (organoleptic score ≥ 2 or gas chromatography VSC ≥ 110 μg/L) or validated questionnaires; studies relying solely on subjective questionnaires were excluded. Inclusion also required extractable data on halitosis prevalence, *H. pylori* infection rates, and halitosis prevalence before and after eradication. Exclusions comprised studies with fewer than 10 patients, guidelines, letters, reviews, abstracts without full text, or insufficient data. Two investigators independently assessed full texts, resolving disagreements through discussion and consensus. All included studies reported *H. pylori* prevalence rather than incidence, as infection status was assessed at a single time point or at baseline, and no study prospectively tracked new infections over time.

### 2.3 Quality Assessment

Methodological quality of the included studies was assessed using design‐specific tools: the Newcastle–Ottawa Scale (NOS) for cohort studies and the Agency for Healthcare Research and Quality (AHRQ) checklist for cross‐sectional studies. The NOS evaluates selection, comparability, and outcome (0–9 points), with scores ≥ 7 indicating high quality. The AHRQ checklist evaluates 11 items (0–11 points), with scores ≥ 9 indicating high quality.

### 2.4 Data Extraction

Using a predefined data extraction form, data were extracted by two investigators (Y.X., J.D.‐Z., T.S.) and confirmed by two others (B.J.‐J., S.L.‐Z.). Disagreements were addressed by consulting a third investigator (H.W.‐X., C.Q.‐X.). The data collected included publication year, first author, sample size, sex, mean age, study type, data source, and intervention measures.

### 2.5 Statistical Analysis

Based on study design, the included studies were systematically categorized into four analytical groups to examine the bidirectional relationship between *H. pylori* infection and halitosis: (1) *H. pylori* prevalence in halitosis versus non‐halitosis populations, (2) halitosis prevalence in *H. pylori* ‐positive versus negative populations, (3) halitosis resolution rates following successful versus unsuccessful eradication therapy in *H. pylori* ‐infected patients, and (4) halitosis prevalence before and after *H. pylori* eradication in infected individuals. All statistical analyses were performed using Stata version 12.0 (StataCorp). Study heterogeneity was evaluated using Cochran's *Q* test (significance threshold *p* < 0.1) and *I* ^2^ statistics (indicating heterogeneity when *I* ^2^ > 50%). Depending on heterogeneity, either fixed‐effects or random‐effects models were applied for meta‐analysis. For analyses with zero events in one or more cells, a continuity correction of 0.5 was added to each cell of the 2 × 2 table to enable calculation of the odds ratio (OR). Deek's funnel plot asymmetry test was used to assess publication bias, with statistical significance set at *p* < 0.05 for all inferential analyses. This comprehensive approach ensured a robust evaluation of both the epidemiological association and therapeutic impact between *H. pylori* infection and halitosis.

## 3 Results

A total of 154 potential studies were identified through electronic database searches, of which 101 were excluded due to duplication, conference abstracts, reviews, or commentaries. Among the remaining 53 articles, 29 were excluded from the meta‐analysis due to sample sizes of fewer than 10 participants, insufficient data, or non‐English literature. The final 24 studies were deemed eligible for further analysis [11, 12, 13, 14, 15, 16, 17, 18, 19, 20, 21, 22, 23, 24, 25, 26, 27, 28, 29, 30, 31, 32, 33, 34]. These included 13 retrospective studies, 9 prospective cohort studies, and 2 cross‐sectional studies. Figure 1 displays the literature screening flowchart and subgroup classification design. Table 1 summarizes the main characteristics of the included studies. Quality assessment results are presented in Supplementary Table 1. The 24 studies were rated as moderate to high quality. Supplementary Table 2 provides a study‐by‐outcome matrix showing the contribution of each included study to the four subgroup analyses. Supplementary Table 3 contains the extracted 2 × 2 data for each included study. This meta‐analysis includes a total of 5872 participants (Group 1: 2305, Group 2: 2389, Group 3: 376, Group 4: 802).

**FIGURE 1:**
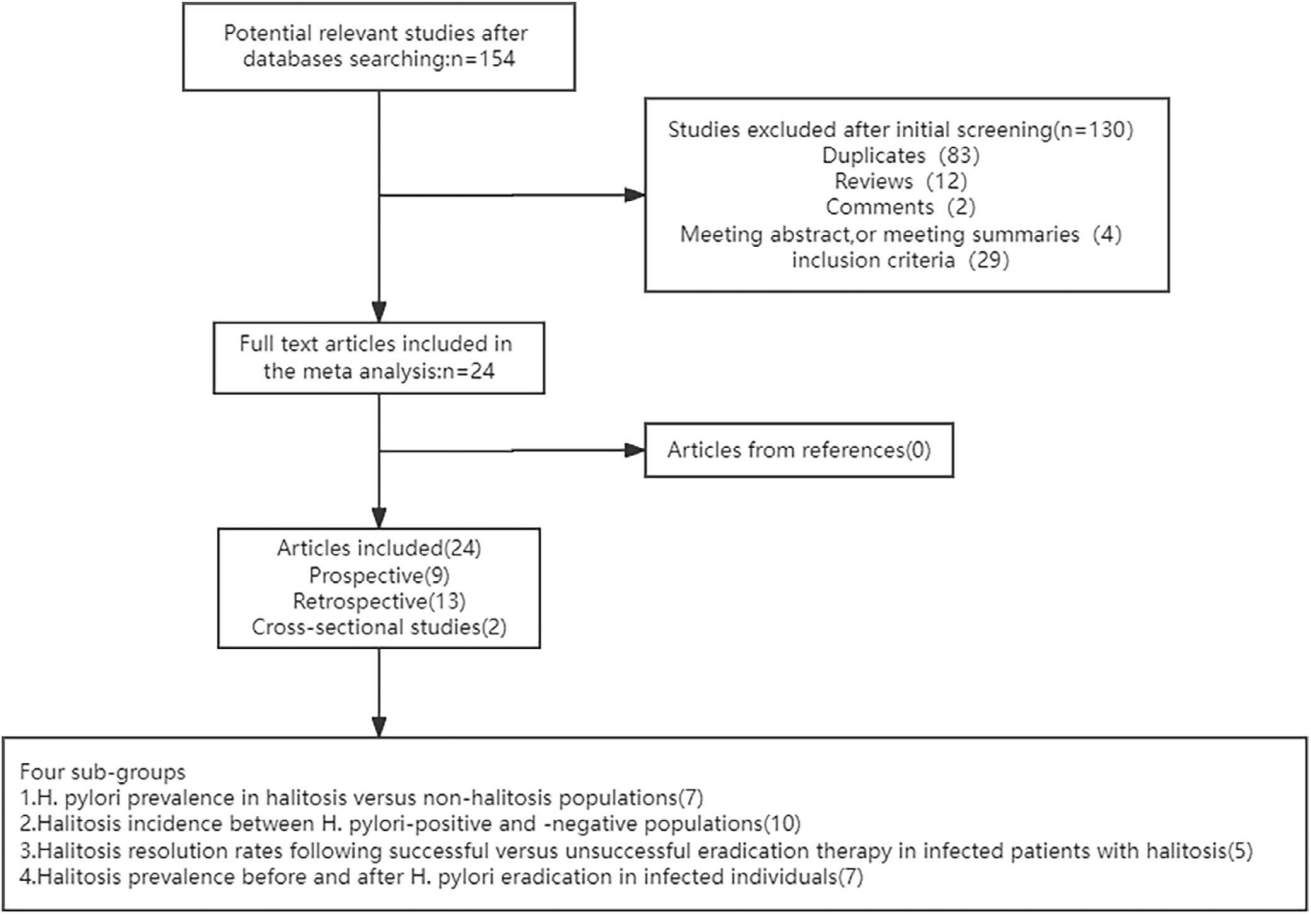
Literature screening flowchart and subgroup classification design.

**TABLE 1:** Characteristics of the studies included in this meta‐analysis.

First author	Publication year	Country	Study type	Case number	Sex (M/F)	Mean age	Halitosis test	*H. pylori* test (location)	Eradication therapy of *H. pylori*
Ierardi	1998	Italy	Prospective	58	29/29	45.3	Halimeter/organoleptic	UBT/histology (stomach)	Double‐drug for 2 weeks (omeprazole + amoxicillin), and Triple‐drug for 10 days in case of eradication failure (omeprazole + clarithromycin + metronidazole)
Gasbarrini	1998	Italy	Retrospective	116	50/66	35 ± 11	Questionnaire	UBT (stomach)	——
Gasbarrini	1999	Italy	Prospective	31	18/13	39 ± 12	Questionnaire	UBT (stomach)	Triple‐drug for 1 week for IDDM patients (pantoprazol + amoxicillin + clarithromycin)
Shashidhar	2000	USA	Prospective	32	13/19	11	Questionnaire	RUT/histology (stomach)	Triple‐drug for 2 weeks (lansoprazol + amoxicillin + clarithromycin)
Werdmuller	2000	The Netherlands	Retrospective	404	188/216	50 (*H. pylori*+) 46 (*H. pylori*−)	Questionnaire	Histology, culture and gram stain, RUT, ELISA (stomach)	——
Hoshi	2002	Japan	Retrospective	80	34/46	44.7 (*H. pylori*+) 33.8 (*H. pylori*−)	Organoleptic	UBT (stomach)	——
Serin	2003	Turkey	Prospective	148	69/79	38.2	Questionnaire	histology (stomach)	Triple‐drug for 2 weeks (omeprazole + amoxicillin + clarithromycin)
Candelli	2003	Italy	Retrospective	121	65/56	14.8 ± 5.6	Questionnaire	UBT (stomach)	——
Adler	2005	Argentina	Retrospective	124	40/84	56.5	Halimeter	PCR (stomach)	——
Katsinelos	2007	Greece	Prospective	18	10/8	40.7 ± 10.9	Questionnaire	UBT/histology (stomach)	Triple‐drug for 10 days (omeprazole + amoxicillin + clarithromycin), and quadruple‐drug for 10 days in case of eradication failure (omeprazole + bismuth + metronidazole + tetracycline)
Suzuki	2008	Japan	Retrospective	326	130/196	45.3 ± 15	Gas chromatography	PCR (saliva)	——
Lee	2009	Korea	Prospective	88	39/49	47.7 ± 10.4	Halimeter	UBT (stomach)	Triple‐drug for 1 week or Red ginseng for 10 weeks
Tangerman	2012	The Netherlands	Retrospective	78	7/4阳性 18/20	60 (*H. pylori*+) 52.9 (*H. pylori*−)	Organoleptic	CLO‐test/histology/culture (stomach)	——
Dore	2012	Italy	Cross‐sectional	1741	Unknown	6–15	Questionnaire	ELISA (stomach)	——
Yilmaz	2012	Turkey	Retrospective	108	54/54	8	Organoleptic	Stool antigen test (stomach)	——
HajiFattai	2015	Iran	Retrospective	44	20/22	34.29 ± 13.71	Organoleptic test	RUT (stomach)	——
Zaric	2015	India	Retrospective	98	42/56	19–78	Organoleptic test	Nestde‐PCR	Triple‐drug for 1 week (amoxicillin + clarythromycin + pantoprazol)
Aksit	2017	Turkey	Retrospective	100	47/53	10.22 ± 3.44	Organoleptic and the BANA test.	PCR (dental biofilm and saliva)	——
Suzuki	2020	Japan	Prospective	12	Unknown	59.5	Organoleptic and gas chromatograph	RUT (stomach)	Triple‐drug for 1 week (lansoprazole + amoxicillin + clarithromycin) and (lansoprazole + amoxicillin + metronidazole) in case of eradication failure
Kudo	2022	Japan	Prospective	77	21/56	64.2 ± 11.5	Total gas detector system	RUT/UBT/histology/serum antibody	Triple‐drug for 1 week (PPI + amoxicillin + clarithromycin/metronidazole)
Zhou	2024	China	Retrospective	574	259/315	45.3	Questionnaire	UBT (stomach)	——
Chen	2024	China	Prospective	100	45/55	33.37 ± 6.35	Organoleptic	UBT (stomach)	Quadruple‐drug for 2 weeks (rabeprazole + bismuth + amoxicillin + clarithromycin)
Karakas	2024	Japan	Cross‐sectional	543	302:241	3.92	Questionnaire	ELISA (feces)	——
Yang	2025	China	Retrospective	108	44/64	38.74 ± 8.42	Organoleptic	UBT (stomach)	——

### 3.1 Group 1 (*n* = 7): *H. pylori* Prevalence in Halitosis vs. Non‐Halitosis Populations

Figure 2 shows an overall OR = 3.42 (95% CI: 1.13, 10.33), indicating that the *H. pylori* infection rate was significantly higher in the halitosis group compared with the non‐halitosis group, with statistical significance. Heterogeneity metrics (*I* ^2^ = 89.9%, *p* = 0.000) revealed substantial heterogeneity across studies. A random‐effects model was employed to account for this heterogeneity and minimize its impact on the pooled estimate. The variability in *H. pylori* detection methods, testing sites, broad age range of subjects, and geographic distribution may introduce confounding factors, considering regional variations in prevalence rates and strain virulence.

**FIGURE 2:**
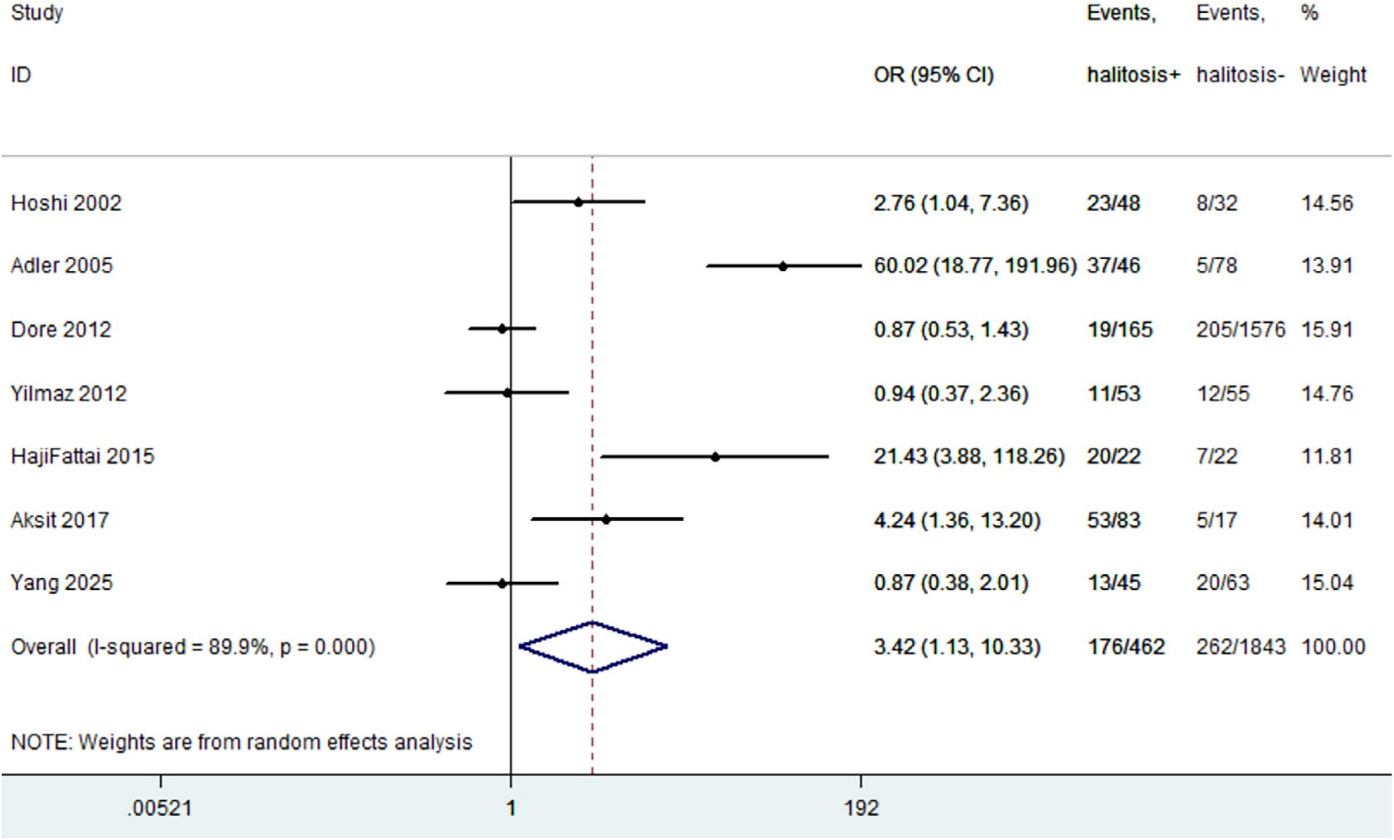
*H. pylori* prevalence in halitosis versus non‐halitosis populations.

### 3.2 Group 2 (*n* = 10): Halitosis Prevalence Between *H. pylori* ‐Positive and ‐Negative Populations

Figure 3 shows an overall OR = 1.91 (95% CI: 1.28, 2.86), demonstrating that the prevalence of halitosis in the *H. pylori* ‐positive group is significantly higher than in the *H. pylori* ‐negative group (*p* < 0.05), suggesting that *H. pylori* infection may be a risk factor for halitosis. Heterogeneity analysis revealed *I* ^2^ = 65.6% (*p* = 0.002), indicating moderate heterogeneity among studies (*I* ^2^ > 50% and *p* < 0.05). A random‐effects model was applied to mitigate the impact of heterogeneity. Heterogeneity may arise from local oral factors and gastrointestinal comorbidities, which could independently influence the occurrence of halitosis and introduce bias into effect size estimation.

**FIGURE 3:**
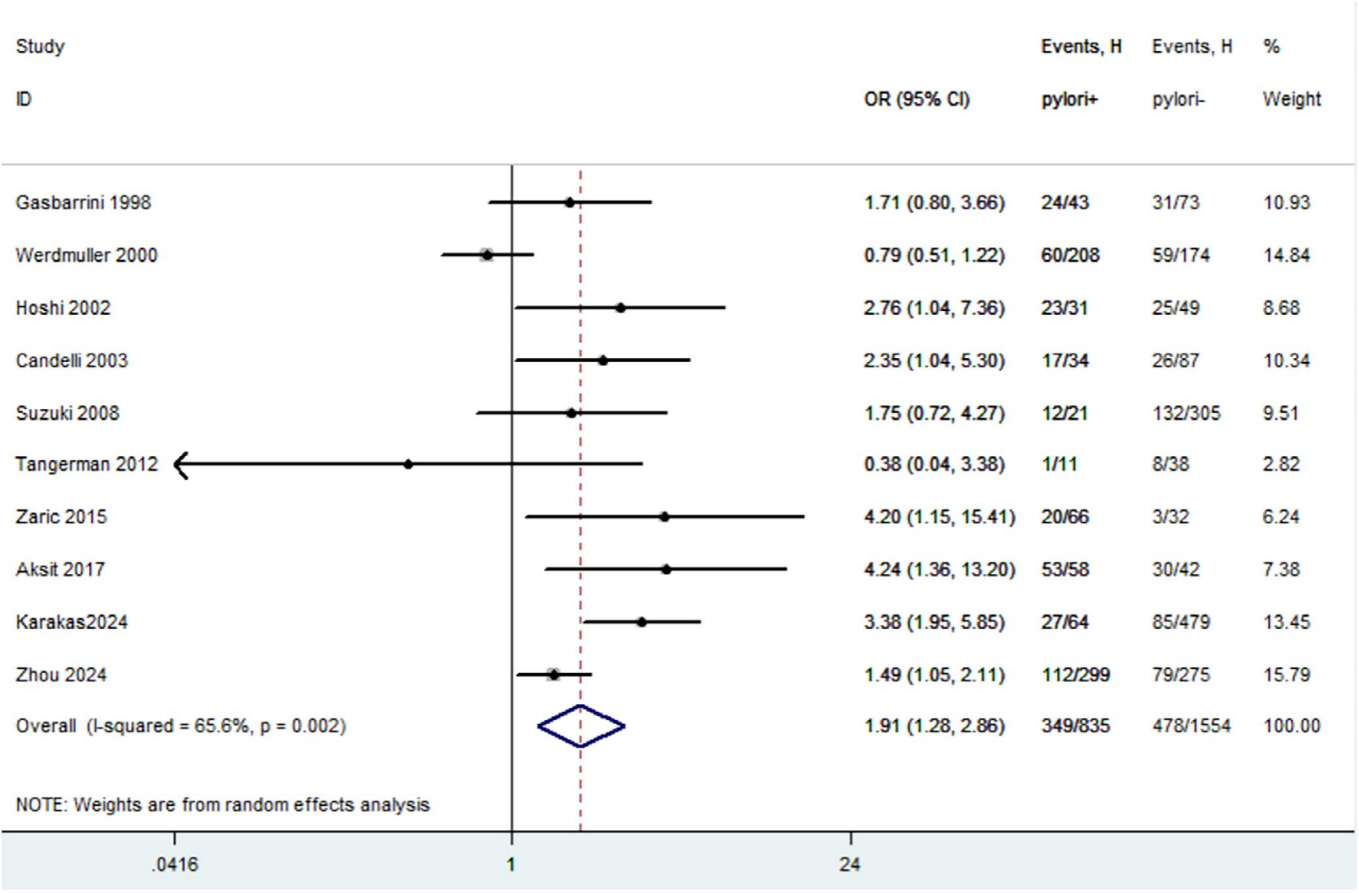
Halitosis prevalence in *H. pylori* ‐positive versus ‐negative populations.

### 3.3 Group 3 (*n* = 5): Halitosis Resolution Rates Following Successful vs. Unsuccessful Eradication Therapy in Infected Patients With Halitosis

Figure 4 shows an overall OR = 0.17 (95% CI: 0.04, 0.73), indicating a significantly lower halitosis rate in the *H. pylori* eradicated group compared with the unsuccessful eradication group. The event was defined as the presence of halitosis after treatment; an OR < 1 favors successful eradication. This suggests that *H. pylori* eradication may reduce the risk of halitosis. Heterogeneity analysis revealed *I* ^2^ = 77.2% (*p* = 0.002), indicating substantial heterogeneity across the studies. A random‐effects model was employed to mitigate the impact of this heterogeneity. Variability in *H. pylori* eradication regimens, lack of standardization in defining eradication success, differences in halitosis assessment timing, and potential confounding from oral *H. pylori* colonization or other causes of halitosis contribute to the observed heterogeneity.

**FIGURE 4:**
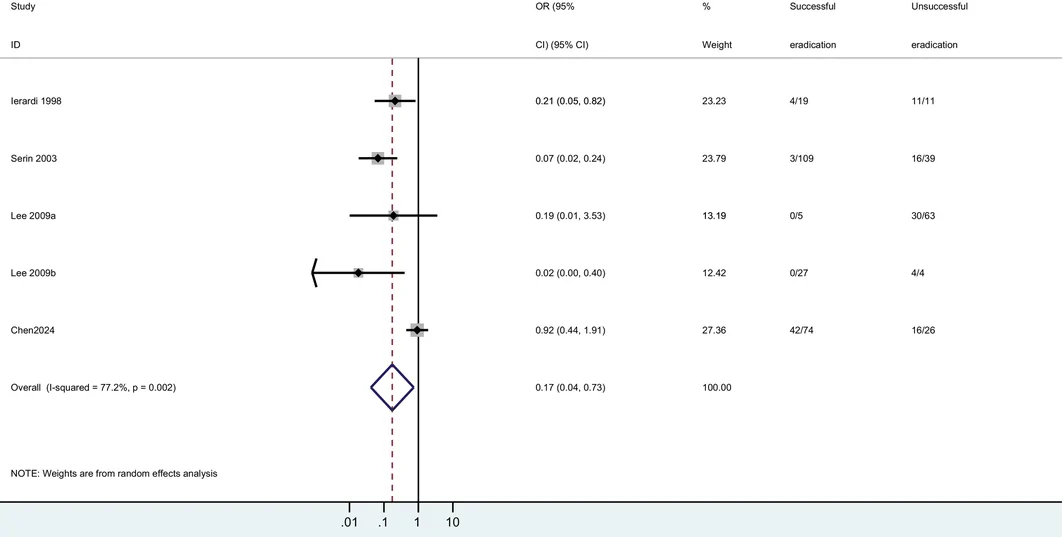
Halitosis resolution rates following successful versus unsuccessful eradication therapy in infected patients with halitosis.

### 3.4 Group 4 (*n* = 7): Halitosis Prevalence Before and After *H. pylori* Eradication in Infected Individuals

Figure 5 demonstrates an overall OR = 4.39 (95% CI: 1.99, 9.68). The event was defined as the presence of halitosis after treatment; an OR > 1 therefore indicates higher odds of halitosis before eradication than after eradication. These results indicate that halitosis prevalence was significantly lower following *H. pylori* eradication compared with pre‐eradication levels. Heterogeneity analysis revealed *I* ^2^ = 76.7% (*p* = 0.000), suggesting substantial heterogeneity among studies. A random‐effects model was applied to minimize its influence. All studies in this group used a self‐controlled before‐after design without parallel control groups, which could not exclude time‐dependent confounders, such as improvements in oral hygiene during the study period. Additionally, variations in follow‐up duration further increased the uncertainty of the results.

**FIGURE 5:**
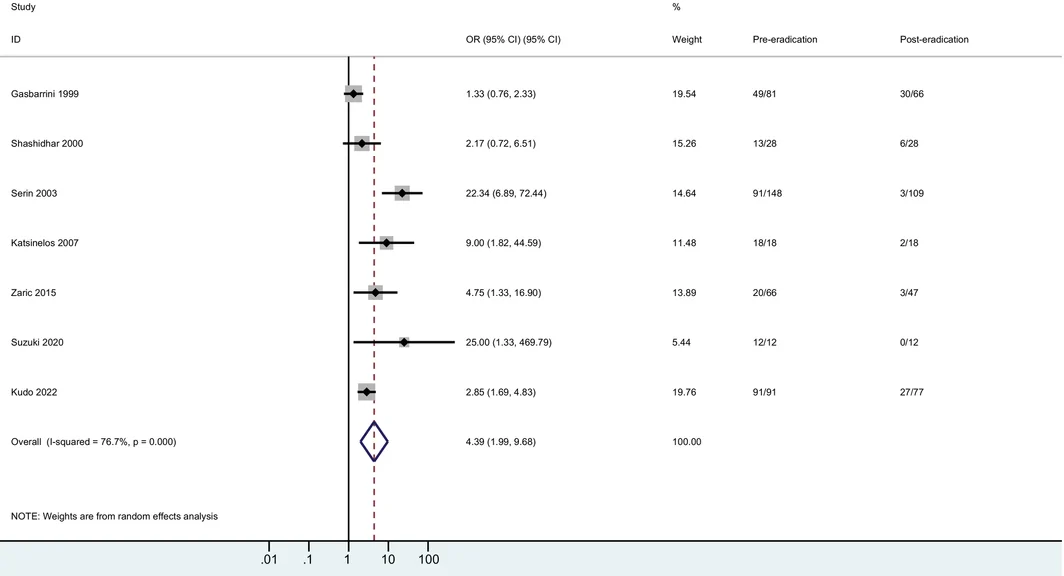
Halitosis prevalence before and after *H. pylori* eradication in infected individuals.

## 4 Discussion

Halitosis, a prevalent clinical symptom, affects approximately 10%–30% of adults in the United States and EU, and 30%–60% in developing countries, significantly impairing quality of life [2]. Its etiology is multifactorial, encompassing oral diseases, gastrointestinal disorders, and systemic conditions [1, 6]. *H. pylori* , a Gram‐negative bacterium that colonizes the stomach, is a well‐established cause of gastritis, peptic ulcers, gastric adenocarcinoma, and gastric MALT lymphoma [35]. Recent studies suggest a potential link between *H. pylori* infection and halitosis. Building upon Dou's 2016 meta‐analysis that demonstrated a significant correlation between *H. pylori* infection and halitosis [10], this study has incorporated updated evidence through May 2025, with a larger sample size of 5872 cases, thereby enhancing statistical power and minimizing random error. The findings further support an observational association between *H. pylori* infection and halitosis, providing clinical support for *H. pylori* screening and eradication therapy in patients with refractory halitosis and no apparent oral or otolaryngological pathology.

As research progresses, numerous studies have sought to elucidate the mechanisms by which *H. pylori* contributes to halitosis. Tonzetich et al. identified the formation of odorous VSCs, such as H_2_S and CH_3_SH, by bacteria in the oral cavity—especially within the tongue coating—as a primary cause of oral malodor [7, 36, 37]. In 2009, Lee et al. found significantly higher VSC concentrations in the oral cavity of *H. pylori* ‐positive patients compared with *H. pylori* ‐negative patients [23]. Gastric *H. pylori* can metabolize urea to produce ammonia and VSCs, or indirectly contribute to halitosis by triggering gastric acid reflux, which alters the oral microenvironment [12, 22, 28]. The high urease activity of *H. pylori* breaks down urea into ammonia and carbon dioxide. Ammonia entering the oral cavity elevates pH, facilitating the proliferation of anaerobic bacteria, which in turn produce VSCs—the primary chemical markers of halitosis [38]. Additionally, *H. pylori* infection can lead to gastric disorders, such as delayed gastric emptying, abnormal gastric acid secretion, and gastroesophageal reflux. Reflux materials may emit foul odors or alter the oral bacterial flora, while inflammatory mediators generated by gastric inflammation can also influence the oral microenvironment [39]. Notably, the relationship between *H. pylori* infection, GERD, and halitosis is complex. Some studies have reported an inverse association between *H. pylori* and GERD, possibly due to *H. pylori* ‐induced gastric atrophy reducing acid secretion [40]. Meanwhile, GERD itself is a recognized cause of halitosis through regurgitation of gastric contents [41]. This apparent paradox suggests that the association between *H. pylori* and halitosis may not be simply mediated by GERD. In our analysis, GERD was not consistently adjusted for in the included primary studies, which we have acknowledged as a limitation. *H. pylori* can also colonize the oral cavity, where oral biofilms provide an optimal environment for its survival, including suitable pH, temperature, and microaerobic conditions. Moreover, oral *H. pylori* can directly produce ammonia and VSCs through urease activity. Its presence in the oral cavity may also serve as a “reservoir” for gastric reinfection, complicating long‐term halitosis management [42, 43].

Currently, the primary treatment for halitosis centers on oral hygiene interventions, but there is no consensus regarding eradication therapy for *H. pylori* ‐positive patients. In 2003, Serin et al. studied 148 *H. pylori* ‐positive patients and observed changes in halitosis rates before and after eradication therapy, finding that 97.2% of those with successful eradication experienced complete resolution of halitosis symptoms [18]. Recent research has increasingly focused on the impact of *H. pylori* eradication on halitosis relief. A 2022 study by Kudo et al. demonstrated that patients with significant halitosis before treatment showed a marked reduction in symptoms post‐eradication, with improvements persisting up to 2 years after treatment, confirming the long‐term benefits of successful eradication in halitosis management [31]. The present study conducted a longitudinal analysis of halitosis rates before and after successful *H. pylori* eradication, alongside a cross‐sectional comparison between successfully eradicated and unsuccessful eradication subgroups. The results indicated an 83% reduction in halitosis prevalence (OR = 0.17, 95% CI: 0.04–0.73), with the pre‐eradication risk of halitosis being 4.4 times higher than post‐eradication (OR = 4.39, 95% CI: 1.99–9.68). These findings highlight the significant clinical value of *H. pylori* eradication in preventing or treating halitosis, particularly in refractory cases with concurrent *H. pylori* infection [31]. However, some have suggested that the reduction in oral malodor may be attributed to the effects of antibiotics on the oral microbiota. Yet, a 2020 study by Suzuki et al. showed that while antibiotic treatment altered the composition of the salivary bacterial community, it returned to baseline levels within 7 weeks. The improvement in halitosis was not linked to a direct antibiotic effect on oral bacteria, as post‐eradication bacterial composition did not differ significantly from pre‐eradication, and the total bacterial count in the oral cavity showed no substantial change [30]. This finding, however, should be interpreted with caution. The comparison between successful and unsuccessful eradication in Group 3 was observational rather than randomized, and may be confounded by factors such as antibiotic adherence, regimen choice, drug resistance, follow‐up duration, and baseline disease severity. These factors were not systematically controlled in the included studies, and may have influenced the observed treatment effects.

Although multiple clinical studies confirm that successful *H. pylori* eradication significantly improves halitosis symptoms, therapeutic efficacy exhibits individual variability. Meta‐analyses indicate persistent halitosis in some patients post‐eradication. Potential mechanisms for this include: (1) transient suppression of the oral microbiota during eradication therapy, followed by recolonization by bacteria such as *Porphyromonas gingivalis* , which continue to produce VSCs [44]; (2) a 2020 meta‐analysis demonstrating a significantly higher prevalence of gastroesophageal reflux in the eradication group compared with controls (RR = 1.54), with gastroesophageal reflux disease correlating with halitosis (OR = 2.24), and a severity‐dependent escalation of risk [45, 46]; and (3) persistent oral infections (e.g., periodontitis, dental caries, tongue coating accumulation) left untreated during eradication therapy [47]. Therefore, a comprehensive evaluation of halitosis etiology is crucial in clinical practice, incorporating both oral treatments and screening for systemic diseases when necessary.

This systematic review and meta‐analysis offers several key strengths. By incorporating 24 studies (total sample size: 5872) published up to 2025, it provides the most current evidence on the association between *H. pylori* infection and halitosis. The sample size notably exceeds that of prior meta‐analyses [10], establishing this as the largest contemporary dataset on the subject. Additionally, the inclusion of studies on *H. pylori* eradication therapy strengthens the evidence base, offering valuable insights for managing refractory halitosis in *H. pylori* ‐infected patients. Methodologically, rigorous criteria for disease diagnosis, study selection, and data extraction enhance the reliability of the conclusions.

However, this meta‐analysis has certain limitations. The restriction to English‐language studies may have led to the omission of research from regions with high *H. pylori* prevalence (e.g., China), resulting in underrepresentation of data from these areas and potential selection bias. Therefore, the pooled estimates should be interpreted with caution in a global context. Future research should include multilingual evidence to improve geographical representation. The included studies exhibited heterogeneity in sample sources, detection methods, and treatment protocols, which could affect result accuracy. However, the limited number of studies in some subgroups precluded further subgroup or sensitivity analyses to explore the sources of heterogeneity. Additionally, most included studies were observational and did not systematically adjust for important confounders such as periodontal disease, tongue coating, oral hygiene, smoking, reflux disease, functional dyspepsia, diabetes, age, sex, or recent antibiotic use. Thus, our findings reflect an observational association rather than a causal relationship. Future prospective studies with larger sample sizes, standardized diagnostic criteria, and rigorous adjustment for these confounders are needed to establish causality and identify which patient subgroups might benefit most from *H. pylori* eradication therapy.

In conclusion, the current evidence presented here confirms an observational association between *H. pylori* infection and halitosis, with eradication therapy showing potential in alleviating oral malodor. However, due to the observational nature of most included studies and lack of adjustment for important confounders, these findings should be interpreted as associative rather than causal.

## Funding

This work was supported by the Young Elite Sponsorship Program of Shandong Provincial Medical Association under Grant Number: 2024‐GJ‐0131.

## Ethics Statement

This meta‐analysis used only published literature and did not involve human subjects, patient data, or animal experiments. Therefore, ethical approval and informed consent were not required.

## Conflicts of Interest

The authors declare no conflicts of interest.

## Supporting information


**Data S1:** PRISMA 2009 checklist.

**Supplementary Table 1.** Quality assessment results.

**Supplementary Table 2.** Study‐by‐outcome matrix.

**Supplementary Table 3.** Extracted 2 × 2 data for each included study.

## Data Availability

The data supporting this meta‐analysis are from previously published studies. All extracted data are presented in this article and its Supplementary Files. The original studies are accessible via PubMed, EMBASE, MEDLINE, and Web of Science, and are cited in the references. No new primary data were generated.
