# Supplementary material for: Halitosis and Helicobacter pylori Infection: A Systematic Review and Meta‐Analysis
Source: JGH Open. 2026 Oct 11;10(10):e70483. doi: 10.1002/jgh3.70483 (PMC13655304; doi:10.1002/jgh3.70483)
Supplement: Supplementary file 2 [file JGH3-10-e70483-s002.docx]

| Study | Group 1 | Group 2 | Group 3 | Group 4 |
| --- | --- | --- | --- | --- |
| Ierardi 1998 |  |  | √ |  |
| Gasbarrini 1998 |  | √ |  |  |
| Gasbarrini 1999 |  |  |  | √ |
| Shashidhar 2000 |  |  |  | √ |
| Werdmuller 2000 |  | √ |  |  |
| Hoshi 2002 | √ | √ |  |  |
| Serin 2003 |  |  | √ | √ |
| Candelli 2003 |  | √ |  |  |
| Adler 2005 | √ |  |  |  |
| Katsinelos 2007 |  |  |  | √ |
| Suzuki 2008 |  | √ |  |  |
| Lee 2009a |  |  | √ |  |
| Lee 2009b |  |  | √ |  |
| Tangerman 2012 |  | √ |  |  |
| Dore 2012 | √ |  |  |  |
| Yilmaz 2012 | √ |  |  |  |
| HajiFattai 2015 | √ |  |  |  |
| Zaric 2015 |  | √ |  | √ |
| Aksit 2017 | √ | √ |  |  |
| Suzuki 2020 |  |  |  | √ |
| Kudo 2022 |  |  |  | √ |
| Zhou 2024 |  | √ |  |  |
| Chen 2024 |  |  | √ |  |
| Karakas 2024 |  | √ |  |  |
| Yang 2025 | √ |  |  |  |

Supplementary Table 2,Study-by-outcome

In Group 3, Lee (2009) contributed two independent subgroup estimates (Lee 2009 a and Lee 2009 b), which were treated as separate data entries in the meta-analysis and are counted individually toward the group total. These two subgroups are derived from the same original study.
