# Supplementary material for: Halitosis and Helicobacter pylori Infection: A Systematic Review and Meta‐Analysis
Source: JGH Open. 2026 Oct 11;10(10):e70483. doi: 10.1002/jgh3.70483 (PMC13655304; doi:10.1002/jgh3.70483)
Supplement: Supplementary file 3 [file JGH3-10-e70483-s003.docx]

| Study | Q1 | Q2 | Q3 | Q4 | Q5 | Q6 | Q7 | Q8 | Q9 | Q10 | Q11 | Total  (max 11) | Quality level |
| --- | --- | --- | --- | --- | --- | --- | --- | --- | --- | --- | --- | --- | --- |
| Dore 2012 | 1 | 1 | 0 | 1 | 1 | 1 | 0.5 | 1 | 0 | 1 | 1 | 8.5 | M |
| Karakas 2024 | 1 | 1 | 1 | 1 | 1 | 1 | 0.5 | 0.5 | 0 | 1 | 1 | 9 | H |

Supplementary Table 1. Quality assessment of included cross‑sectional studies using the AHRQ checklist.

Annotations (11 items):

1. Research question/objective clearly stated

2. Study population from same source with clear inclusion criteria

3. Sample size justification or calculation provided

4. Standardized data collection methods used

5. Outcome measures clearly defined, valid, reliable

6. Exposure measures clearly defined, valid, reliable

7. Important confounding factors controlled for

8. Response rate reported

9. Missing data described and handled appropriately

10. Statistical analysis appropriate

11. Conclusions consistent with results and limitations discussed

Scoring:

1 = Fully meets criterion

0.5 = Partially meets criterion

0 = Does not meet criterion / Not reported

Quality level:

9–11 points: High quality(H)

7–8.5 points: Moderate quality(M)

≤6.5points: Low quality(L)

| Study | Selection (max 4) | Comparability (max 2) | Exposure/Outcome (max 3) | Total (max 9) | Quality Level |
| --- | --- | --- | --- | --- | --- |
| Ierardi 1998 | 3 | 2 | 3 | 8 | H |
| Gasbarrini 1998 | 4 | 1 | 2 | 7 | H |
| Gasbarrini 1999 | 4 | 1 | 3 | 8 | H |
| Shashidhar 2000 | 3 | 1 | 3 | 7 | H |
| Werdmuller 2000 | 4 | 2 | 2 | 8 | H |
| Hoshi 2002 | 3 | 1 | 2 | 6 | M |
| Serin 2003 | 3 | 2 | 2 | 7 | H |
| Candelli 2003 | 4 | 2 | 2 | 8 | H |
| Adler 2005 | 2 | 2 | 2 | 6 | M |
| Katsinelos 2007 | 3 | 2 | 3 | 8 | H |
| Suzuki 2008 | 3 | 1 | 2 | 6 | M |
| Lee 2009 | 4 | 0 | 3 | 7 | H |
| Tangerman 2012 | 3 | 2 | 2 | 7 | H |
| Yilmaz 2012 | 3 | 2 | 2 | 7 | H |
| HajiFattahi 2015 | 2 | 2 | 2 | 6 | M |
| Zaric 2015 | 3 | 2 | 3 | 8 | H |
| Aksit 2017 | 3 | 2 | 2 | 7 | H |
| Suzuki 2020 | 3 | 1 | 3 | 7 | H |
| Kudo 2022 | 3 | 1 | 3 | 7 | H |
| Zhou 2024 | 4 | 2 | 3 | 9 | H |
| Chen 2024 | 3 | 1 | 3 | 7 | H |
| Yang 2025 | 4 | 2 | 3 | 9 | H |

Supplementary Table 1. Quality assessment of included cohort studies using the Newcastle‑Ottawa Scale (NOS).

Quality level:

7–9 points: High quality(H)

5–6 points: Moderate quality(M)

0–4 points: Low quality(L)
