# Supplementary material for: Halitosis and Helicobacter pylori Infection: A Systematic Review and Meta‐Analysis
Source: JGH Open. 2026 Oct 11;10(10):e70483. doi: 10.1002/jgh3.70483 (PMC13655304; doi:10.1002/jgh3.70483)
Supplement: Supplementary file 4 [file JGH3-10-e70483-s004.docx]

| Study | halitosis +/HP+ | halitosis +/HP- | halitosis -/HP+ | halitosis -/HP- |
| --- | --- | --- | --- | --- |
| Hoshi 2002 | 23 | 25 | 8 | 24 |
| Adler 2005 | 37 | 9 | 5 | 73 |
| Dore 2012 | 19 | 146 | 205 | 1371 |
| Yilmaz 2012 | 11 | 42 | 12 | 43 |
| HajiFattai 2015 | 20 | 2 | 7 | 15 |
| Aksit 2017 | 53 | 30 | 5 | 12 |
| Yang 2025 | 13 | 32 | 20 | 43 |
| Gasbarrini 1998 | 24 | 31 | 19 | 42 |
| Werdmuller 2000 | 60 | 59 | 148 | 115 |
| Candelli 2003 | 17 | 26 | 17 | 61 |
| Suzuki 2008 | 12 | 132 | 9 | 173 |
| Tangerman 2012 | 1 | 8 | 10 | 30 |
| Zaric 2015 | 20 | 3 | 46 | 29 |
| Zhou 2024 | 112 | 79 | 187 | 196 |
| Karakas 2024 | 27 | 85 | 37 | 394 |

| Study | Success / Halitosis+ | Success / Halitosis- | Failure / Halitosis+ | Failure / Halitosis- |
| --- | --- | --- | --- | --- |
| Ierardi 1998 | 4 | 15 | 11 | 0 |
| Serin 2003 | 3 | 106 | 16 | 23 |
| Lee 2009a | 0 | 5 | 30 | 33 |
| Lee 2009b | 0 | 27 | 4 | 0 |
| Chen 2024 | 42 | 32 | 16 | 10 |

| Study | Pre / Halitosis+ | Pre / Halitosis- | Post / Halitosis+ | Post / Halitosis- |
| --- | --- | --- | --- | --- |
| Gasbarrini 1999 | 49 | 32 | 30 | 36 |
| Shashidhar 2000 | 13 | 15 | 6 | 22 |
| Serin 2003 | 91 | 57 | 3 | 106 |
| Katsinelos 2007 | 18 | 0 | 2 | 16 |
| Zaric 2015 | 20 | 46 | 3 | 44 |
| Suzuki 2020 | 12 | 0 | 0 | 12 |
| Kudo 2022 | 91 | 0 | 27 | 50 |

Supplementary Table 3. Extracted 2×2 data for each included study
